# Regulatory role of long non-coding RNA UCA1 in signaling pathways and its clinical applications

**DOI:** 10.3892/ol.2021.12665

**Published:** 2021-03-19

**Authors:** Zhaoping Liu, Yanyan Wang, Shunling Yuan, Feng Wen, Jing Liu, Liheng Zou, Ji Zhang

**Affiliations:** 1 Department of Rheumatology, The First Affiliated Hospital, University of South China, Hengyang, Hunan 421001, P.R. China; 2 Department of Hematology, The First Affiliated Hospital, University of South China, Hengyang, Hunan 421001, P.R. China; 3 Molecular Biology Research Center and Center for Medical Genetics, School of Life Sciences, Central South University, Changsha, Hunan 410078, P.R. China; 4 Department of Clinical Laboratory, Shenzhen Traditional Chinese Medicine Hospital, Shenzhen, Guangdong 518033, P.R. China

**Keywords:** lncRNA, UCA1, signaling pathway, diagnosis, prognosis

## Abstract

Long non-coding RNA metastasis-associated urothelial carcinoma associated 1 (*UCA1*) plays a pivotal role in various human diseases. Its gene expression is regulated by several factors, including transcription factors, chromatin remodeling and epigenetic modification. *UCA1* is involved in the regulation of the PI3K/AKT, Wnt/β-catenin, MAPK, NF-κB and JAK/STAT signaling pathways, affecting a series of cellular biological functions, such as cell proliferation, apoptosis, migration, invasion and tumor drug resistance. Furthermore, *UCA1* is used as a novel potential biomarker for disease diagnosis and prognosis, as well as a target for clinical gene therapy. The present review systematically summarizes and elucidates the mechanisms of upstream transcriptional regulation of *UCA1*, the regulatory role of *UCA1* in multiple signaling pathways in the occurrence and development of several diseases, and its potential applications in clinical treatment.

## 1. Introduction

The whole genome sequencing of eukaryotes has confirmed that ~93% of the DNA in the human genome can be transcribed into RNA (1). Notably, only 2% of mRNAs encode proteins and the remaining 98% are non-coding RNAs (1). Among the different types of non-coding transcripts, long non-coding RNAs (lncRNAs) have attracted great interest. lncRNAs do not contain any extended open reading frames (ORFs) and are >200 nucleotides in length (2). They have been identified as indispensable regulators in a variety of physiological and pathological processes, including epigenetic inheritance, cell cycle, post-transcriptional regulation, translation and chromatin modification (3). In addition, lncRNAs participate in the regulation of cell proliferation, differentiation, apoptosis, invasion, migration and tumor drug resistance (4). lncRNAs play important roles in the occurrence and development of various diseases, particularly malignant tumors (5,6). Previous studies have demonstrated that lncRNAs function as either oncogenes or tumor suppressors, and are involved in the regulation of tumorigenesis and progression of various tumors (5,6).

**Figure 1.**
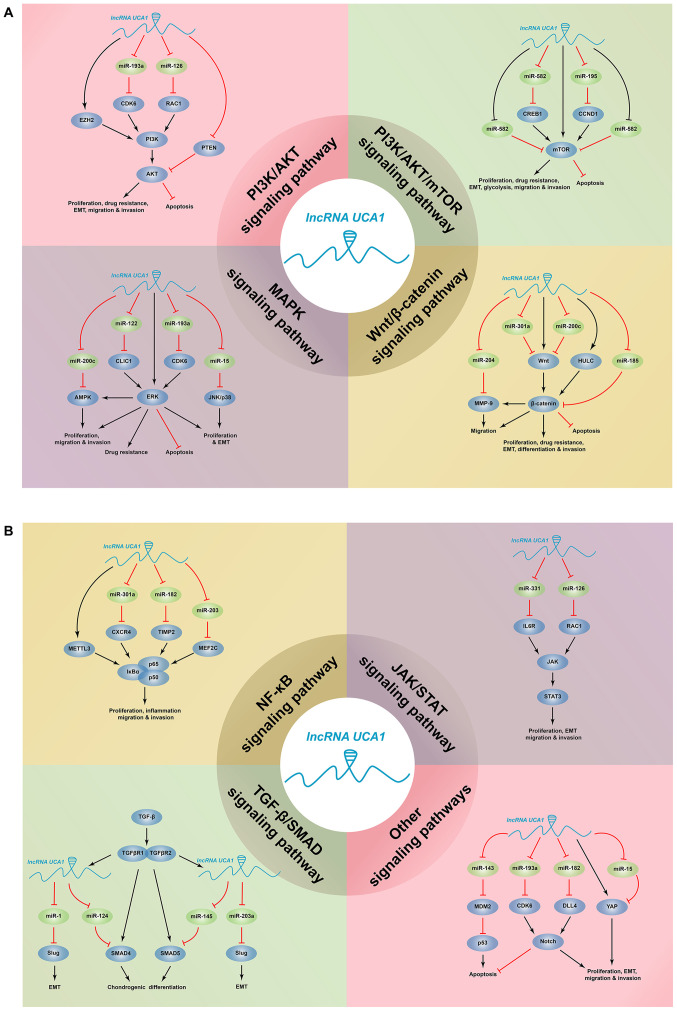
Signaling pathways in diseases mediated by *UCA1*. *UCA1* regulates key signaling pathways by acting as a competitive endogenous RNA, thus affecting biological activities, such as cell proliferation, apoptosis, migration, invasion and drug resistance in cancers and non-cancerous diseases. UCA1, urothelial carcinoma associated 1; CCND1, cyclin D1; CDK6, cyclin-dependent kinase 6; CLIC1, chloride intracellular channel 1; CREB1, cAMP response element-binding protein 1; CXCR4, C-X-C chemokine receptor type 4; DLL4, Delta-like 4; EZH2, enhancer of zeste homolog 2; HULC, highly upregulated in liver cancer; MEF2C, myocyte enhancer factor 2C; METTL3, methyltransferase-like 3; MMP-9, matrix metalloproteinase-9; mTOR, mammalian target of rapamycin; RAC1, RAS-related C3 botulinus toxin substrate 1; TGF-β, transforming growth factor-β; TIMP2, tissue inhibitor of metalloproteinase-2; YAP, yes-associated protein; miR, microRNA; EMT, epithelial-to-mesenchymal transition; lncRNA, long non-coding RNA.

Urothelial carcinoma associated 1 (*UCA1*) was initially identified in bladder cancer by high-throughput RNA sequencing, and is associated with the progression of bladder cancer (7). *UCA1* has been demonstrated to be highly expressed in several human tumors, such as gastric cancer (GC) and cholangiocarcinoma, which is closely associated with tumor-node-metastasis (TNM) stage, depth of invasion, vascular invasion, lymph node metastasis, overall survival (OS) and relapse-free survival (RFS) (8–10). In addition, *UCA1* is involved in regulating cell proliferation, apoptosis, migration, invasion and drug resistance (11). Several studies have reported that *UCA1* participates in the regulation of multiple cellular signaling pathways at transcriptional, post-transcriptional and epigenetic levels (Fig. 1) (12,13). The present review discusses the molecular mechanisms and clinical potential of UCA1 in the regulation of signaling pathways and transcription in human diseases.

## 2. Overview of *UCA1*

In 2006, *UCA1* was demonstrated to be highly specific and sensitive in the diagnosis of bladder cancer, particularly in patients with superficial G2-G3 (7). Furthermore, *UCA1* is considered to be the most specific gene for bladder transitional cell carcinoma (TCC) (7). *UCAI* exhibits a notable differential diagnostic function in several urinary tract diseases without TCC (7).

The *UCA1* gene contains three exons and two introns, and is located on the positive strand of human chromosome 19p13.12 (7). Notably, the *UCA1* sequence contains multiple termination sequences, without any conservative long ORFs (14,15). Initially, the full-length cDNA of *UCA1* was identified as 1.4 kb (7). However, recent studies have demonstrated that *UCA1* has three different isoforms (1.4, 2.2 and 2.7 kb) (16,17). Currently, the 1.4 kb isoform has attracted great interest (14). In addition to its associations with non-cancerous diseases, including systemic lupus erythematosus (SLE), diabetic nephropathy (DN) and Parkinson's disease (PD) (18–20), *UCA1* is also involved in the progression of different types of cancer, including bladder cancer, colorectal cancer (CRC) and hepatocellular carcinoma (HCC) (21–23). Furthermore, overexpression of *UCA1* is closely associated with clinicopathological characteristics, including poor prognostic factors, such as TNM stage, vascular invasion and lymph node metastasis (8,24,25).

**Figure 2.**
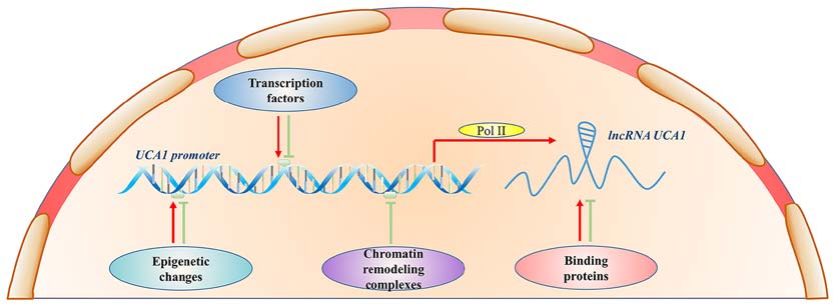
Regulatory mechanisms of *UCA1*. Transcription factors, epigenetic changes, chromatin remodeling complexes and binding proteins positively or negatively regulate *UCA1* expression. UCA1, urothelial carcinoma associated 1; lncRNA, long non-coding RNA; Pol II, RNA polymerase II.

Increasing evidence suggest that *UCA1* functions as an oncogene, which plays an important role in the tumorigenesis and development of different types of cancer, including papillary thyroid carcinoma (26), pancreatic cancer (PC) (27) and lung adenocarcinoma (28). *UCA1* expression is regulated by several factors, such as transcription factors, chromatin remodeling and epigenetic modification (Fig. 2) (29–31). Furthermore, *UCA1* contains microRNA (miRNA/miR) binding sites that regulate the expression of target genes through the sponging of miRNAs (Table I) (8,11,24).

## 3. Upstream regulation of *UCA1* expression

As presented in Table II, upstream regulators of *UCA1* include transcription factors, chromatin remodeling complexes, epigenetic changes and binding proteins (29,31–33). The core promoter of the *UCA1* gene can bind with several transcription factors, such as CCAAT/enhancer binding protein α (C/EBPα) (32), C/EBPβ (34), E26 transformation-specific transcription factor 2 (35), specificity protein 1 (36), MYB (37) and E1A binding protein p300 (EP300) (38). These transcription factors interact with the promoter to upregulate *UCA1* expression, and SND1 can upregulate *UCA1* expression through transcriptional activator *MYB* and promote 5-fluorouracil (5-FU)-induced apoptosis of HCC cells (37). Similarly, macrophage-derived chemokine CCL18 upregulates *UCA1* expression through transcription factor EP300 in osteosarcoma (38). In addition, the combination of hyaluronic acid and CD44 stimulates the signal transduction of PI3K and AKT, resulting in phosphorylation of C/EBPα, which binds to the promoter of the *UCA1* gene to induce transcriptional activation of *UCA1*, resulting in migration and invasion of HSC-3 cells in human head and neck squamous cell cancer (39).

AT-rich interaction domain 1A (ARID1A) is one of the major members of SWItch/Sucrose non-fermentable chromatin remodeling complexes, which is often found to be loss-of-function mutations in different types of cancer, such as non-small cell lung cancer (NSCLC) (40), breast cancer (BC) (41) and pancreatic ductal adenocarcinoma (PDAC) (42). ARID1A inhibits *UCA1* expression by regulating chromatin access of transcription factor C/EBPα (30). It has been confirmed that the CAPERα/TBX3 complex directly inhibits *UCA1* transcription and promotes cancer cell senescence by regulating chromatin structure (43). Furthermore, the AT-rich sequence binding protein 1 suppresses *UCA1* expression by closing the chromatin structure of the *UCA1* promoter region in BC (29). Notably, epigenetic modification participates in regulating *UCA1* expression. S-adenosylmethionine inhibits *UCA1* transcription by increasing DNA methyltransferase or decreasing DNA demethylase (31). The stability of *UCA1* is notably improved due to the formation of functional ribonucleoprotein complexes between hnRNPI and *UCA1* (44). Conversely, *UCA1* interacts with insulin-like growth factor 2 mRNA-binding protein 1 and is downregulated due to its reduced stability (45).

Bone morphogenetic protein 9 (BMP9), a member of the BMP family, belongs to the transforming growth factor-β (TGF-β) superfamily. TGF-β is a well-known inducer of epithelial-to-mesenchymal transition (EMT) (33,46). BMP9 or TGF-β can upregulate *UCA1* expression to promote invasion and metastasis of cancer cells, including BC and GC cells (33,46). In addition, TGF-β inactivates the Hippo pathway by regulating the complex of transcriptional co-activator with PDZ binding motif/yes-associated protein (YAP) and transcriptional enhancer TEA domain (TEAD), which subsequently upregulates *UCA1* expression and promotes the migration and invasion of BC cells (47). Some clinical trials have reported that metformin exhibits anticancer potential and can be used as an adjuvant for cancer prevention or an AMPK activator for cancer treatment (48,49). Metformin can decrease endometrial hyperplasia via the *UCA1*/miR-144/TGF-β1/AKT signaling pathway (50). In addition, metformin suppresses the migratory ability of trophoblast cells by regulating the signal transduction pathway of *UCA1*/miR-204/MMP-9 (51). Notably, lncRNA GAS8-AS1 inhibits migration and invasion of osteosarcoma cells by downregulating *UCA1* expression (52). Furthermore, up-frameshift protein 1, palmitic acid, cancer-associated fibroblasts and hepatitis B virus X protein can also regulate *UCA1* expression and affect the proliferation of cancer cells, including HCC, GC and CRC cells (53–56).

## 4. Signaling pathways regulated by *UCA1*

### 

#### PI3K/AKT signaling pathway

The PI3K/AKT signaling pathway is involved in important physiological activities, including cell proliferation, invasion and metastasis, and is closely associated with cancer (16,35), DN (19), SLE (18), myocardial fibrosis (57) and PD (20). Recently, the regulatory mechanisms of lncRNAs in the PI3K/AKT pathway and their effects on diseases have attracted great interest (58–60).

It has been reported that *UCA1* can promote malignant phenotypes of PDAC by activating the AKT signaling pathway (16). This process includes the promotion of cell proliferation, invasion, EMT, inhibition of apoptosis and enhancement of 5-FU resistance (16). In bladder cancer (35) and SLE (18), *UCA1* is highly expressed and promotes cell proliferation by mediating the PI3K/AKT signaling pathway. *UCA1* knockdown inhibits proliferation and migration of glioma cells through miR-193a-mediated downregulation of cyclin-dependent kinase 6 (CDK6), and inactivates the PI3K/AKT pathway by decreasing the expression levels of phosphorylated PI3K and AKT proteins (61). In acute myeloid leukemia (AML), *UCA1* can be used as an endogenous sponge to compete with miR-126, which in turn suppresses activation of the PI3K/AKT signaling pathway by inhibiting the expression of RAS-related C3 botulinus toxin substrate 1 (RAC1) (62).

Recent studies have demonstrated that enhancer of zeste homolog 2 (EZH2) can promote cell cycle progression by affecting the PI3K/AKT pathway or the expression levels of cyclins (63–65). EZH2 can also be post-translationally modified by phosphorylation of AKT (66). *UCA1* directly interacts with *EZH2* in GC and enhances EZH2 expression, which in turn activates the AKT/GSK3β/cyclin D1 (CCND1) axis to increase cell proliferation (36). However, *UCA1* suppresses p27Kip1/CDK2 signal transduction and promotes cell proliferation and tumorigenesis by recruiting EZH2 (56). In PC (67) and BC (44), *UCA1* promotes the proliferation of cancer cells by inhibiting *p27* expression. *UCA1* can also inhibit the level of cAMP response element-binding (CREB) protein, and regulate the cell cycle of bladder cancer cells via the PI3K-dependent pathway (68). A study has reported that *UCA1* impairs the binding of brahma-related gene 1 (*BRG1*) to the *p21* promoter and the chromatin remodeling activity of *BRG1* to accelerate the proliferation of bladder cancer cells (69). Cisplatin and gemcitabine resistance in bladder cancer cells is mediated by *UCA1*-CREB-miR-196a-5p paradigm (70).

*UCA1* can inhibit PTEN and promote p-AKT expression to induce the proliferation of osteosarcoma cells (71). Notably, p-PTEN is a phosphatase, and PTEN is a negative regulator of PI3K. Dephosphorylation of PTEN can decrease activation of AKT and block its downstream signal transduction (71). *In vivo* studies have demonstrated that *UCA1* functions via the PI3K/AKT signaling pathway (19,48,72). In the Sprague-Dawley rat model of DN, inhibition of *UCA1* decreases renal pathological damage, improved renal function and decreased inflammation in DN rats by suppressing the PI3K/AKT signaling pathway (19). In addition, downregulation of *UCA1* expression can ameliorate the damage of dopaminergic neurons by inhibiting the PI3K/AKT signaling pathway to decrease oxidative stress and inflammation in PD (20). Thus, *UCA1* participates in the regulation of the PI3K/AKT signaling pathway by affecting EZH2, CDK6, RAC1 and PTEN, which promote cell proliferation, invasion, metastasis and drug resistance, and inhibit apoptosis in diseases (36,61,62,71).

Several studies have demonstrated that mammalian target of rapamycin (mTOR) is the core component downstream of the PI3K/AKT pathway (73–75). lncRNAs participate in cell proliferation, apoptosis, invasion and energy metabolism by regulating the mTOR signaling pathway (73). It has been reported that upregulation of *UCA1* expression confers tamoxifen resistance in BC cells, partly by activating the mTOR signaling pathway (74). In addition, *UCA1* interacts with mTOR to inhibit *p27* and miR-143 expression; however, the expression levels of Kirsten rat sarcoma viral oncogene homolog and CCND1 significantly increase, which results in the proliferation, EMT and metastasis of CRC cells (55). Notably, it has been demonstrated that *UCA1* increases *CREB1* expression by acting as a competitive endogenous RNA (ceRNA) of miR-582, thus promoting EMT via the CREB1-mediated mTOR pathway, which results in osteosarcoma metastasis (72). In cardiomyocytes, *UCA1* decreases miR-122 and miR-143 expression, and regulates their downstream mTOR signaling pathway to inhibit oxygen-glucose deprivation (OGD) or hypoxia/reoxygenation (H/R)-induced apoptosis and injury (76,77). *UCA1* knockdown can also regulate signal transduction of mTOR via miR-200c and miR-195, and inhibit the proliferation and invasion of hemangioma cells and microvascular endothelial cells (78). Furthermore, bladder cancer cells preferentially metabolize glucose by aerobic glycolysis, known as the Warburg effect (79). *UCA1* promotes glycolysis and upregulates hexokinase 2 expression via the mTOR-STAT3/miR-143 pathway in bladder cancer cells (79). Taken together, these findings suggest that *UCA1* can directly or indirectly activate the mTOR signaling pathway by serving as a regulator of miR-582 and miR-195, and affect the expression of proteins, such as CREB1 and CCND1, to promote cell proliferation, drug resistance and inhibit apoptosis.

#### Wnt/β-catenin signaling pathway

The Wnt/β-catenin signaling pathway is a developmental signal-transduction pathway. Activation of this pathway can result in the proliferation, invasion, metastasis and differentiation of cancer cells, ultimately inducing tumorigenesis (80). The canonical Wnt pathway activates gene transcription through β-catenin accumulation in the cytoplasm and translocation to the nucleus (80).

Several studies have demonstrated that *UCA1* promotes cell proliferation and EMT in osteosarcoma (38), glioma (81) and papillary thyroid carcinoma (82) by activating the Wnt/β-catenin signaling pathway. In addition, the Wnt/β-catenin signaling pathway is closely associated with chemoresistance (12,81,83). *UCA1* induces chemoresistance to cisplatin and temozolomide in glioma and decreases the sensitivity of BC cells to tamoxifen via the Wnt/β-catenin signaling pathway (81,83). Notably, *UCA1* can also increase cisplatin resistance of bladder cancer cells by activating Wnt signaling in a Wnt6-dependent manner (12).

Previous studies have reported that *UCA1* regulates the Wnt/β-catenin signaling pathway via miR-200c and miR-185-5p, and affects the proliferation and EMT in hemangioma (78) and melanoma cells (84), respectively. In osteosarcoma, *UCA1* knockdown inhibits miR-301a expression and induces the silencing of C-X-C chemokine receptor type 4 (*CXCR4*), thus decreasing cell proliferation and metastasis (85). In addition, downregulation of miR-301a expression decreases the levels of Wnt3a, Wnt5a and β-catenin proteins, which in turn inhibits the Wnt/β-catenin signaling pathway (85). *UCA1* regulates matrix metalloproteinase-9 expression, which is a downstream gene of Wnt/β-catenin (86), via miR-204 and increases trophoblast cells migration (51). Notably, enhanced *UCA1* expression upregulates the expression of highly upregulated in liver cancer (*HULC*) by inhibiting *HULC* promoter methylation, and upregulates β-catenin by promoting β-catenin promoter-enhancer chromatin DNA looping formation, which in turn promotes the malignant transformation of hepatocyte-like cells (87). Collectively, these findings suggest that *UCA1* is involved in tumorigenesis via the Wnt/β-catenin signaling pathway by regulating miRNAs, such as miR-204 and miR-301a, and lncRNAs, such as *HULC*.

#### MAPK signaling pathway

The MAPK pathway participates in the regulation of cell proliferation, invasion, metastasis, apoptosis and differentiation, and is closely associated with the occurrence of various diseases such as PDAC (16), melanocytes (13) and acute myocardial infarction (76). Its functional pattern predominantly involves phosphorylating nuclear transcription factors, cytoskeletal proteins and enzymes (88). The MAPK signaling pathway includes four pathways: The ERK, P38, SAPK/JNK and ERK5/BMK1 pathways. As an important regulatory gene, *UCA1* can participate in the regulation of the MAPK signaling pathway (88).

A previous study demonstrated that *UCA1* promotes the proliferation, migration and invasion of PDAC cells, and decreases apoptosis and increases 5-FU resistance by activating the ERK signaling pathway (16). In addition, *UCA1* negatively regulates the CREB/MITF/melanogenesis axis by suppressing the ERK and JNK signaling pathways in melanocytes, thus inhibiting the expression of melanogenesis-related genes in melanocytes (13).

*UCA1* knockdown activates the AMPK signaling pathway by upregulating a series of miRNAs (61,76,78,89). In addition, *UCA1* knockdown regulates AMPK signal transduction via miR-200c and suppresses the proliferation, migration and invasion of hemangioma cells (78). Furthermore, in acute myocardial infarction, *UCA1* decreases OGD aroused cell apoptosis and injury by downregulating miR-122 expression and suppressing its downstream JNK/p38 MAPK signaling pathway (76). Knockdown of *UCA1* inactivates the MAPK signaling pathway by downregulating *CDK6* expression mediated by miR-193a, which decreases the proliferation and promotes the apoptosis of glioma cells (61). In cholangiocarcinoma, *UCA1* upregulates chloride intracellular channel 1 (CLIC1) expression by inhibiting miR-122 expression and activating the ERK/MAPK signaling pathway to promote the metastasis of malignant cells (89). In addition, *UCA1* promotes the viability and EMT of TPC-1 cells by competing with miR-15a and activating the Hippo and JNK signaling pathways in human thyroid cancer (90). Taken together, these findings suggest that *UCA1* activates the MAPK signaling pathways by regulating CLIC1 and CDK6 expression, inducing cell proliferation, migration and drug resistance, and inhibiting apoptosis.

#### Nuclear factor-kappa B (NF-κB) signaling pathway

NF-κB is an extensively expressed multi effect transcription factor. It is a heterotrimer composed of p50, p65 and IκB (91). The NF-κB signaling pathway is activated by several factors, including extracellular stimulation and transcription factors (91). Activated NF-κB signaling pathway mediates several biological processes, such as cell proliferation, tumor metastasis and inflammation (91).

*UCA1* can activate the NF-κB signaling pathway through inflammatory cytokines like IL6 (92) and transcription factors like MEF2C (93). Several studies have demonstrated that the NF-κB signaling pathway can be regulated by *UCA1* (85,94). In osteosarcoma, inhibition of *UCA1* downregulates miR-301a expression and decreases *CXCR4* expression, thus suppressing cell proliferation and metastasis (85). Furthermore, miR-301a positively regulates the phosphorylation of IκB and p65 proteins and activates the NF-κB signaling pathway (85). *UCA1* knockdown exerts an anticancer effect on GC cells by rescuing miR-182 expression to activate the NF-κB and PI3K/AKT/GSK3β signaling pathways (94). In HCC, the synergism between *UCA1* and inflammatory cytokine IL6 promotes *SUV39H1* expression. Notably, methyltransferase-like 3, which binds to *SUV39H1* mRNA, is also upregulated. Excessive *SUV39H1* expression can increase the expression of tri-methylation of histone 3 lysine 9 trimethylation (H3K9me3) (92). Notably, under inflammatory conditions, H3K9me3 induces phosphorylation of NF-κB, resulting in the malignant transformation of hepatocyte-like stem cells (92). This synergism between *UCA1* and IL6 can aggravate the malignant transformation of hepatocyte-like stem cells via the NF-κB signaling pathway (92). In addition, *UCA1* regulates the inflammatory responses in epilepsy by regulating the miR-203-mediated myocyte enhancer factor 2C/NF-κB signaling pathway (93).

#### JAK/STAT signaling pathway

The JAK/STAT signaling pathway is a common pathway for various cytokines and growth factors to transmit signals within cells (95). It mediates several biological responses, including cell proliferation, differentiation, migration, apoptosis and immune regulation (95). Previous studies have demonstrated that lncRNAs play a regulatory role in the JAK/STAT signaling pathway (96,97).

*UCA1* promotes the development of cancer cells by activating the JAK/STAT signaling pathway, including multiple myeloma (MM) (98), AML (62) and pre-eclampsia (99). *UCA1* activates the JAK2/STAT3 pathway via the miR-331-3p/IL6R axis in MM, and regulates cell proliferation and apoptosis (98). In AML, *UCA1* competes with miR-126 as an endogenous sponge. miR-126 suppresses activation of the JAK/STAT signaling pathway by decreasing RAC1 expression, thus inhibiting cell proliferation, migration and invasion (62). Notably, *UCA1* exerts opposite effects on the regulation of the JAK/STAT signaling pathway in some non-cancerous cells (99,100). A study has reported that *UCA1* expression is upregulated in trophoblast cells of pre-eclampsia pregnancy, and it can inhibit the invasion and proliferation of trophoblast cells by downregulating JAK2 expression (99). In addition, overexpression of *UCA1* inhibits activation of the JAK/STAT signaling pathway, which in turn inhibits activation of astrocytes in temporal lobe epilepsy (100). It is suggested that *UCA1* promotes IL6R and RAC1 expression to stimulate the JAK/STAT signaling pathway, which increases cell proliferation and invasion in several tumors, while *UCA1* exerts opposing effects in some non-cancerous cells (62,98).

#### TGF-β/SMAD signaling pathway

The TGF-β superfamily is composed of secretory polypeptide molecule TGF-β, activins, inhibins and BMPs, and participates in various biological activities, such as cell invasion and migration (101). The TGF-β superfamily is involved in cartilage and bone formation, inflammation, regulation of endocrine functions, and formation and development of tumors (101). SMADs are important molecules for intracellular TGF-β signal transduction (101).

Previous studies have demonstrated that EMT induced by TGF-β1 is an important reason for the invasion and migration of cancer cells (46,102). The *UCA1*/miR-124 axis regulates TGF-β1-induced EMT and invasion of tongue cancer cells (46). In addition, *UCA1* promotes the proliferation of MM cells by targeting TGF-β (102). SMAD4 and SMAD5, two members of SMADs, are important elements involved in the TGF-β pathway (103). It has been confirmed that *UCA1* regulates the TGF-β signaling pathway via the miR-145-5p/SMAD5 and miR-124-3p/SMAD4 axes and promotes chondrogenic differentiation of human bone marrow mesenchymal stem cells (103). Notably, *UCA1* competitively binds with miR-1 and miR-203a to upregulate the expression of Slug, a downstream effector of TGF-β (104), and subsequently regulates EMT in glioma cells (104) and BC cells (33).

#### Other signaling pathways

*UCA1* also participates in the regulation of other signaling pathways, including the Notch, Hippo and p53 signaling pathways. In human glioma, *UCA1* knockdown inhibits the proliferation and migration of cells through miR-193a-mediated downregulation of CDK6, and blocks the Notch signaling pathway by decreasing the expression levels of phosphorylated Notch1, Notch2 and Notch3 proteins (61). In renal cell carcinoma, *UCA1* upregulates Delta-like 4 (DLL4) expression by acting as a ceRNA of miR-182-5p, and induces the EMT process via the DLL4-mediated Notch pathway, which in turn promotes cell proliferation and migration, and inhibits apoptosis (11). Furthermore, *UCA1* can form a ribonucleoprotein complex with Mps one binder kinase activator-1, large tumor suppressor 1 and YAP, which decreases the phosphorylation of YAP (105). Dephosphorylated YAP translocates into the nucleus and combines with TEAD protein, inhibiting the key proteins of the Hippo signaling pathway and promoting proliferation, migration and invasion of PC cells (105). Conversely, *UCA1* activates the Hippo signaling pathway by downregulating miR-15a expression and increasing cell viability and EMT in human thyroid cancer (90). In cardiomyocytes, *UCA1* inhibits H/R-induced apoptosis by decreasing miR-143 expression and suppressing its downstream p53 signaling pathway (77). Furthermore, *UCA1* can regulate the p53 signaling pathway via miR-143 and MDM2; the Notch signaling pathway via CDK6 and DLL4 or regulate YAP and other signaling pathways, which promotes cell proliferation, EMT, migration and invasion, and inhibits cell apoptosis (11,61,105).

## 5. Clinical applications of *UCA1*

Several studies have reported that *UCA1* has potential clinical applications (106–109). Currently, there are three common clinical applications of *UCA1*: Acts as a diagnostic biomarker (110,111), prognostic biomarker (112,113) and therapeutic target (87). The use of *UCA1* as a therapeutic target for reversing drug resistance has been extensively investigated (22,83).

### 

#### UCA1 as a diagnostic biomarker

Early diagnosis is beneficial to the prognosis of diseases. Poor prognosis is mainly attributed to the metastasis of cancer cells, drug resistance and tumor recurrence, which are closely associated with late diagnosis (114). Thus, it is important to improve the early diagnostic rate of patients. Recently, several studies have demonstrated that lncRNAs have great potential to be used as biomarkers for disease diagnosis, including *UCA1* (115–117).

*UCA1* is highly specific and easy to be detected in serum, plasma and urine. Liquid biopsy is becoming a novel method for disease detection (72,118). In laryngeal squamous cell carcinoma (110), NSCLC (111), osteosarcoma (72) and HCC (118), serum *UCA1* levels of patients are higher than that of the healthy control population. Notably, *UCA1* secreted by exosomes into the serum of patients can promote the development of prostate cancer (PCa) (114). In addition, serum *UCA1* expression is significantly higher in patients with HCC than those with benign liver disease, which helps to distinguish the two groups (119). In non-cancerous diseases, *UCA1* can limit the inflammatory responses in epilepsy (93), and *UCA1* expression is higher in patients with non-refractory epilepsy than those with refractory epilepsy (120). Notably, combined measurement of *UCA1* levels in serum and plasma can increase the sensitivity and specificity for the diagnosis of some malignancies (119). In addition, *UCA1* expression is higher in the urine of patients with PCa compared with healthy controls (114). Taken together, these findings suggest that *UCA1* has the potential to be used as a biomarker for diagnosis and screening of diseases.

Increasing evidence suggest that the combination of *UCA1* and other lncRNAs may exhibit better diagnostic performance. As a predictor of CRC, the combination of *UCA1*, HOXA transcript at the distal tip and plasmacytoma variant translocation 1 has better diagnostic performance compared with *UCA1* alone, and can be used to screen patients with advanced CRC (115). Another study demonstrated that the combination of *UCA1*, gastric cancer high expressed transcript 1, taurine upregulated gene 1 and *p21*-associated ncRNA DNA damage activated can improve the diagnostic ability and significance of GC (116). Furthermore, when comparing patients with bladder cancer with healthy controls, the combination of *UCA1, circFARSA* and *circSHKBP1* has better diagnostic performance compared with *UCA1* alone (117).

Early differential diagnosis of cancer and precancerous lesions is an important method to decrease the risk of recurrence and improve prognosis (121). A study has demonstrated that the combination of *UCA1*, HOX transcript antisense intergenic RNA, hydatidiform mole associated and imprinted and metastasis-associated lung adenocarcinoma transcript 1 can distinguish between patients with bladder cancer and patients with urocystitis, with a sensitivity and specificity of 95.7% and 94.3%, respectively (121). Collectively, these findings suggest that it is feasible to use *UCA1* as a diagnostic biomarker. However, further studies are required to verify the clinical applications of *UCA1*.

#### UCA1 as a prognostic biomarker

With the rapid growth of disease morbidity and mortality, overall prognosis will become the principal determinant of global public health (122). Surgery is one of the routine therapies for cancer; however, its complications and recurrence affect the prognosis of patients (122). Recent studies have demonstrated that *UCA1* is closely associated with clinicopathological characteristics, including vascular invasion, lymph node metastasis and TNM stage, and can be used as a prognostic biomarker of diseases (24,25,123). However, there is no doubt whether further investigation on the role of *UCA1* as a prognostic marker will contribute to the treatment of cancer.

**Figure 3.**
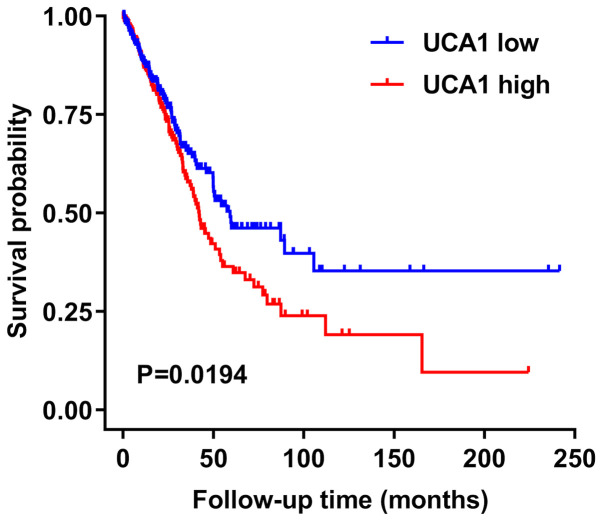
Association between *UCA1* expression and survival time of patients with non-small cell lung cancer, based on The Cancer Genome Atlas data. UCA1, urothelial carcinoma associated 1.

*UCA1* is closely associated with tumor size, histological differentiation, stage of lymph node metastasis, depth of invasion, vascular invasion, OS, RFS and prognostic biomarkers (Table III). It has been reported that in some cancers, such as CRC (24), HCC (23), GC (8), adrenal cortical carcinoma (124) and esophageal cancer (125), survival probability of patients is associated with *UCA1* expression. The Cancer Genome Atlas (https://cancergenome.nih.gov) data for lung adenocarcinoma revealed that the survival probability of patients with NSCLC, with high *UCA1* expression, is unfavorable than those with low *UCA1* expression (Fig. 3). Previous studies have suggested that overexpression of *UCA1* in CRC indicates a large tumor, advanced TNM stage, deeply infiltrated lymph node, positive lymph node metastasis and poor OS (22,24). Other studies have reported that *UCA1* expression in GC tissues and cells is positively associated with TNM stage, lymph node infiltration, lymph node metastasis and OS (8,9). Notably, *UCA1* expression in the plasma of patients with GC significantly decreases following surgery (126). Thus, *UCA1* can be used as an index of postoperative prognosis. In addition, *UCA1* expression is significantly upregulated in SLE, which is positively associated with SLE disease activity index (18). Collectively, these findings suggest that *UCA1* can be used as an independent prognostic factor to monitor the occurrence and development of diseases.

#### UCA1 as a therapeutic target

With a better understanding of the pathogenesis of diseases, several molecules and signal transduction pathways are likely to be suitable for targeted therapy (122). Given that *UCA1* exerts carcinogenic effects associated with several molecules and cascade reactions, it has been confirmed as an ideal therapeutic target (22,70,81). Targeted knockout of *UCA1* can be used to improve radiosensitivity, inhibit cancer metastasis, prevent cancer growth *in vivo*, promote apoptosis and reverse drug resistance of cancer cells (21,37,72). Clinical applications of *UCA1* as a therapeutic target for reversing drug resistance has attracted great interest (21,127). *UCA1* is also known as a cancer upregulated drug resistant gene (87). Previous studies have reported that downregulating *UCA1* expression can reverse drug resistance in cancers, and some drugs for chemotherapy exert an anticancer role by mediating *UCA1* (Table IV), including cisplatin (70,81), tamoxifen (83), paclitaxel (128), 5-FU (22), Adriamycin (129), temozolomide (81), cetuximab (130), trastuzumab (131), imatinib (132), docetaxel (133) and gemcitabine (70). These drugs provide the possibility of chemotherapy-related clinical applications, and studies have demonstrated that *UCA1* knockdown can reverse multidrug resistance in retinoblastoma (127) and bladder cancer (21). In addition, the combination of *UCA1*-targeted therapy and programmed cell death 1 (PD-1) immune checkpoint blockade has a better synergistic effect following CRISPR-Cas9-mediated *UCA1* knockdown (134). However, further studies are required to confirm the therapeutic value of *UCA1* for diseases.

## 6. Conclusions and perspective

*UCA1* can regulate a series of signaling pathways by acting as a ceRNA of several miRNAs, and affect epigenetic, transcriptional and post-transcriptional regulation. It plays a regulatory role in several biological functions, including cell proliferation, apoptosis, migration, invasion and drug resistance. In addition, *UCA1* can be used as a potential biomarker for disease diagnosis and treatment. For some diseases, the combination of *UCA1* and other lncRNAs has demonstrated better diagnostic and screening performance. Strategies, such as CRISPR-Cas9 system or siRNA-mediated knockout, and the combination of *UCA1*-targeted therapy and PD-1 immune checkpoint blockade may be used to transform *UCA1* from basic research into clinical practice.

Recently, although great progress has been made in understanding the biology of *UCA1*, even in its therapeutic applications, several unknown areas in *UCA1* research remain. First, the lack of effective methods to investigate RNA secondary and tertiary structures and nuclear ultrastructure of *UCA1* impedes the study on the composition of its isoforms and tissue-specific regulation (4). Secondly, several studies have confirmed that lncRNAs can promote the drug resistance of cancer cells by regulating their stemness feature (27,135,136,104); however, the mechanism by which *UCA1* affects the drug resistance of cancer stem cells remains unclear. Some lncRNAs may play important regulatory roles in cellular biological processes via multiple pathways (5,6). However, whether binding miRNAs affects the expression or function of *UCA1* remains unclear. In conclusion, *UCA1*, as a well-known lncRNA with great clinical potential, requires increasing comprehension and in-depth study.

## Figures and Tables

**Table I.** Regulation of signaling pathways mediated by UCA1 as a competitive endogenous RNA of miRNAs.

miRNA	Target gene	Signaling pathways	Biological functions	Diseases	(Refs.)
miR-193a	CDK6	PI3K/AKT, ERK/MAPK and Notch	Migration, invasion and apoptosis	Glioma	(61)
miR-126	RAC1	PI3K/AKT and JAK/STAT	Proliferation, migration and invasion	AML	(62)
miR-582	CREB1	PI3K/AKT/mTOR	EMT	Osteosarcoma	(72)
miR-143	HK2	PI3K/AKT/mTOR	Glycolysis	Bladder cancer	(79)
miR-200c	–	PI3K/AKT/mTOR, AMPK and Wnt/β-catenin	Proliferation, migration and invasion	Hemangioma	(78)
miR-185-5p	MMP-9	Wnt/β-catenin	EMT	Melanoma	(84)
miR-122	–	PI3K/AKT/mTOR and JNK/p38 MAPK	Apoptosis	AMI	(76)
miR-122	CLIC1	ERK/MAPK	Metastasis	CCA	(89)
miR-203	MEF2C	NF-κB	Inflammation	Epilepsy	(93)
miR-331-3p	IL6R	JAK2/STAT3	Proliferation and apoptosis	MM	(98)
miR-15a	–	Hippo and JNK/p38 MAPK	Proliferation and EMT	Thyroid cancer	(90)
miR-124	JAG1	Notch	Invasion and EMT	Tongue cancer	(46)
miR-145-5p	SMAD5	TGF-β/SMAD	Chondrogenic differentiation	Osteoarthritis	(103)
miR-124-3p	SMAD4	TGF-β/SMAD	Chondrogenic differentiation	Osteoarthritis	(103)
miR-1	Slug	TGF-β/SMAD	EMT	BC and glioma	(33,104)
miR-203a					
miR-182-5p	DLL4	Notch	Proliferation, migration and apoptosis	Renal cancer	(11)
miR-143	MDM2	PI3K/AKT/mTOR and p53	Apoptosis	AMI	(77)

UCA1, urothelial carcinoma associated 1; miR, microRNA; CDK6, cyclin-dependent kinase 6; AMI, acute myocardial infarction; AML, acute myeloid leukemia; BC, breast cancer; CCA, cholangiocarcinoma; CLIC1, chloride intracellular channel 1; CREB1, cAMP response element-binding protein 1; DLL4, Delta-like 4; EMT, epithelial-to-mesenchymal transition; ERK, extracellular signal-regulated kinase; HK2, hexokinase 2; JAG1, jagged 1; MAPK, mitogen-activated protein kinases; MM, multiple myeloma; MMP-9, matrix metalloproteinase-9; mTOR, mammalian target of rapamycin; PE, pre-eclampsia; RAC1, RAS-related C3 botulinus toxin substrate 1; TGF-β, transforming growth factor-β; -, not available.

**Table II.** Upstream regulation of UCA1 expression.

Type of factor	Regulation	Regulatory factor	Diseases	(Refs.)
Transcription factors	Promotion	C/EBPα	Bladder cancer	(32)
	Promotion	C/EBPβ	Bladder cancer	(34)
	Promotion	Ets-2	Bladder cancer	(35)
	Promotion	SP1	GC	(36)
	Promotion	MYB	HCC	(37)
	Promotion	EP300	Osteosarcoma	(38)
	Promotion	C/EBPα	HNSCC	(39)
Chromatin remodeling complexes	Inhibition	SATB1	BC	(29)
	Inhibition	ARID1A	BC	(30)
	Inhibition	CAPERα/TBX3 repressor complex	UMS	(43)
Epigenetic changes	Inhibition	SAM	HCC	(31)
Binding proteins	Promotion	HnRNPI	BC	(44)
	Inhibition	IMP1	BC	(45)
Others	Promotion	TGF-β	Tongue cancer	(46)
	Promotion	BMP9	BC	(33)
	Promotion	TAZ/YAP and TEAD complexes	BC	(47)
	Inhibition	Metformin	EH	(50)
	Inhibition	Metformin	PE	(51)
	Inhibition	lncRNA GAS8-AS1	Osteosarcoma	(52)
	Inhibition	UPF1	HCC	(53)
	Promotion	PA	GC	(54)
	Promotion	CAFs	CRC	(55)
	Promotion	HBx	HCC	(56)

UCA1, urothelial carcinoma associated 1; ARID1A, AT-rich interaction domain 1A; BC, breast cancer; BMP9, bone morphogenetic protein 9; C/EBP, CCAAT/enhancer binding protein; CAFs, cancer-associated fibroblasts; CRC, colorectal cancer; EH, endometrial hyperplasia; EP300, E1A binding protein p300; ETS-2, E26 transformation-specific transcription factor 2; GC, gastric cancer; HBx, hepatitis B virus X protein; HCC, hepatocellular carcinoma; HNSCC, head and neck squamous cancer; IMP1, insulin-like growth factor 2 mRNA-binding protein 1; PA, palmitic acid; PE, pre-eclampsia; SAM, S-adenosylmethionine; SATB1, special AT-rich sequence binding protein 1; SP1, specificity protein 1; TAZ, transcriptional co-activator with PDZ binding motif; TEAD, transcriptional enhancer TEA domain; TGF-β, transforming growth factor-β; UMS, ulnar-mammary syndrome; UPF1, up-frameshift protein 1; YAP, yes-associated protein.

**Table III.** Role of UCA1 in clinical correlation and prognosis of diseases.

Disease	Upregulation/downregulation	Clinical correlation	(Refs.)
Renal cancer	Upregulation	Degree of differentiation, TNM stage and lymph node metastasis	(11,107)
SLE	Upregulation	SLEDAI	(18)
CRC	Upregulation	Tumor size, tumor stage, lymph node infiltration, lymph node metastasis and OS	(22,24)
HCC	Upregulation	Tumor stage, TNM stage, Microvascular infiltration and OS	(23)
GC	Upregulation	TNM stage, tumor stage, lymph node infiltration, lymph node metastasis and OS	(8,9)
Tongue cancer	Upregulation	TNM staging, lymph node metastasis, lymph node infiltration and OS	(46)
Osteosarcoma	Upregulation	Tumor stage, tumor size and OS	(72)
OSCC	Upregulation	TNM stage, lymph node metastasis and OS	(108)
PTC	Upregulation	Tumor size, tumor stage, lymph node metastasis and OS	(26,82)
MM	Upregulation	OS	(98,102)
PC	Upregulation	Tumor stage and OS	(27,105)
LSCC	Upregulation	Lymph node metastasis and OS	(110)
NSCLC	Upregulation	TNM stage, tumor size and OS	(111)
PCa	Upregulation	Edmondson-Steiner grade, TNM stage and lymph node metastasis	(112,114)
Epilepsy	Upregulation	Classification of epilepsy	(120)
Glioma	Upregulation	TNM stage, tumor size, lymph node metastasis and OS	(109)
Cholangiocarcinoma	Upregulation	TNM stage, tumor stage, lymph node infiltration, lymph node metastasis, RFS and OS	(10)
OC	Upregulation	TNM stage, lymph node metastasis and OS	(128)
NPC	Upregulation	Tumor stage, lymph node metastasis and OS	(113)
ACC	Upregulation	TNM stage and lymph node metastasis	(124)
LUAD	Upregulation	TNM stage, lymph node metastasis, RFS and OS	(28)
Melanoma	Upregulation	Tumor stage and lymph node metastasis	(106)
EC	Upregulation	Degree of differentiation, TNM stage, lymph node metastasis and OS	(125)

UCA1, urothelial carcinoma associated 1; ACC, adrenal cortical carcinoma; CRC, colorectal cancer; EC, esophageal cancer; GC, gastric cancer; HCC, hepatocellular carcinoma; LSCC, laryngeal squamous cell carcinoma; LUAD, lung adenocarcinoma; MM, multiple myeloma; NPC, nasopharyngeal carcinoma; NSCLC, non-small cell lung cancer; OC, ovarian cancer; OS, overall survival; OSCC, oral squamous cell carcinoma; PC, pancreatic cancer; PCa, prostate cancer; PTC, papillary thyroid carcinoma; RFS, relapse-free survival; SLEDAI, systemic lupus erythematosus disease activity index; TNM, tumor-node-metastasis.

**Table IV.** Role of UCA1 in tumor drug resistance.

Cancer type	Drug resistance	Mechanisms	(Refs.)
PDAC	5-FU	UCA1 promotes EMT by activating the AKT and ERK signaling pathways in PDAC cells	(16)
Bladder cancer	Cisplatin/gemcitabine	UCA1 inhibits cisplatin/gemcitabine-induced apoptosis via targeting p27Kip1	(70)
Bladder cancer	Cisplatin	UCA1 upregulates Wnt6 expression	(12)
Bladder cancer	Multidrug resistance	UCA1 inhibits autophagy by upregulating ATG7 expression	(21)
OC	Paclitaxel	UCA1 upregulates ABCB1 expression	(128)
CRC	5-FU	UCA1 upregulates CREB1 expression	(22)
AML	Adriamycin	UCA1 upregulates the HK2 expression	(129)
BC	Tamoxifen	UCA1 activates the mTOR signaling pathway	(74)
BC	Tamoxifen	UCA1 activates the Wnt/β-catenin signaling pathway	(83)
BC	Trastuzumab	UCA1 upregulates YAP1 expression	(131)
Glioma	Cisplatin/temozolomide	UCA1 activates the Wnt/β-catenin signaling pathway	(81)
CRC	Cetuximab	UCA1 in exosomes transmits cetuximab resistance	(130)
CML	Imatinib	UCA1 upregulates MDR1 expression	(132)
PCa	Docetaxel	UCA1 upregulates Sirt1 expression	(133)
Retinoblastoma	Multidrug resistance	UCA1 upregulates STMN1 expression	(127)

UCA1, urothelial carcinoma associated 1; 5-FU, 5-fluorouracil; AML, acute myeloid leukemia; BC, breast cancer; CML, chronic myeloid leukemia; CRC, colorectal cancer; CREB1, cAMP response element-binding protein 1; HK2, hexokinase 2; MDR1, multidrug resistance protein 1; mTOR, mammalian target of rapamycin; OC, ovarian cancer; PCa, prostate cancer; PDAC, pancreatic ductal adenocarcinoma; STMN1, stathmin 1; YAP1, yes-associated protein 1.

## Data Availability

Not applicable.

## Abbreviations

5-FU: 5-fluorouracil
AML: acute myeloid leukemia
ARID1A: AT-rich interaction domain 1A
BC: breast cancer
BMP9: bone morphogenetic protein 9
BRG1: brahma-related gene 1
C/EBPα: CCAAT/enhancer binding protein α
CCND1: cyclin D1
CDK6: cyclin-dependent kinase 6
ceRNA: competitive endogenous RNA
CLIC1: chloride intracellular channel 1
CRC: colorectal cancer
CREB: cAMP response element-binding
CXCR4: C-X-C chemokine receptor type 4
DLL4: Delta-like 4
DN: diabetic nephropathy
EMT: epithelial-to-mesenchymal transition
EP300: E1A binding protein p300
EZH2: enhancer of zeste homolog 2
GC: gastric cancer
H/R: hypoxia/reoxygenation
H3K9me3: histone 3 lysine 9 trimethylation
HCC: hepatocellular carcinoma
HULC: highly upregulated in liver cancer
lncRNA: long non-coding RNA
miRNA/miR: microRNA
MM: multiple myeloma
mTOR: mammalian target of rapamycin
NSCLC: non-small cell lung cancer
OGD: oxygen-glucose deprivation
ORFs: open reading frames
OS: overall survival
PC: pancreatic cancer
PD: Parkinson's disease
PD-1: programmed cell death 1
PDAC: pancreatic ductal adenocarcinoma
RAC1: RAS-related C3 botulinus toxin substrate 1
RFS: relapse-free survival
SLE: systemic lupus erythematosus
TCC: transitional cell carcinoma
TEAD: TEA domain
TGF-β: transforming growth factor-β
TNM: tumor-node-metastasis
UCA1: urothelial carcinoma associated 1
YAP: yes-associated protein
